# Heat Unit Requirements of “Flame Seedless” Table Grape: A Tool to Predict Its Harvest Period in Protected Cultivation

**DOI:** 10.3390/plants10050904

**Published:** 2021-04-30

**Authors:** Francisca Alonso, Fernando M. Chiamolera, Juan J. Hueso, Mónica González, Julián Cuevas

**Affiliations:** 1 Andalusian Institute of Agricultural and Fisheries Research and Training (IFAPA), 04745 La Mojonera, Almería, Spain; paquialonso10@gmail.com; 2 Department of Agronomy, ceiA3, University of Almería, 04120 Almería, Almería, Spain; fmc1984@ual.es; 3 Experimental Station of Foundation Cajamar, 04710 El Ejido, Almería, Spain; juanjosehueso@fundacioncajamar.com (J.J.H.); monicagonzalez@fundacioncajamar.com (M.G.)

**Keywords:** *Vitis vinifera*, growing degree days, base temperature, high threshold temperature, forecasting harvest

## Abstract

Greenhouse cultivation of table grapes is a challenge due to difficulties imposed by their perennial habit and chilling requirements. Despite difficulties, greenhouse cultivation allows ripening long before that in the open field. Nonetheless, for harvesting “Flame Seedless” in the most profitable periods, a cultural practices timetable has to be established. In this context, an estimation of development rate as a function of temperature becomes essential. This work puts forward a procedure to determine “Flame Seedless” threshold temperatures and heat requirements from bud break to ripening. “Flame Seedless” required an average of 1633 growing degree days (GDD) in the open field with a base temperature of 5 °C and an upper threshold temperature of 30 °C. Strikingly, only 1542 GDD were required within the greenhouse. This procedure forecast “Flame Seedless” ripening with an accuracy of three and six days in the open field and greenhouse, improving predictions based on the average number of days between bud break and ripening. The procedure to predict oncoming harvest date was found satisfactory, just four days earlier than the real date. If we used the typical meteorological year instead of the average year, then the prediction was greatly improved since harvest was forecast just one day before its occurrence.

## 1. Introduction

Protected cultivation is a common orientation for the production of out of season vegetables in Southeast Spain, home of the world’s largest concentration of greenhouses. Greenhouse cultivation of fruit trees is less common due to the difficulty imposed by the perennial habit of fruit crops. Nonetheless, fruit crops have been successfully grown in glasshouses since the 17th Century, when orangeries were built in Europe to protect tropical and subtropical crops from the risks of winter frosts [1]. In the case of temperate zone fruit trees and vines, the need to satisfy their chilling requirements for bud break constitutes an additional challenge for managing these crops under greenhouses.

Despite difficulties, the temporary covering of the vines with plastic allows table grapes to be produced well in advance of vines grown in the open field [2,3,4]. Previous research has shown that covering “Flame Seedless” table grape plants with plastic in early December accelerates harvest by one month with respect to plants grown in a nearby vineyard in the open field [5]. This success is explained because the greenhouse provides suitable temperatures early in the season when outdoor conditions are low for bud break and initial shoot growth. The precise climate control achieved in modern greenhouses and the low chilling requirements of “Flame Seedless” table grape thus allow its harvest to be scheduled in early June (Northern Hemisphere) when prices are very high.

The determination of heat requirements of a plant genotype is a useful tool for forecasting its harvest date. A French meteorologist, Alfred Angot, published in the 1880s the units of heat required to ripen grapes in France, Switzerland and Germany, proposing a base temperature of 9 °C [6]. In the first half of the 20th Century, Frederic T. Bioletti adapted those values for their use in California [7] and Winkler and Williams [8] used, in Europe and California, too, a base temperature of 50 °F (10 °C) to determine heat units needed to reach berry maturation of “Tokay” grapes. It was Amerine and Winkler [9] who went on to establish a heat units index (Winkler index) for all wine grape cultivars growing in California using also a base temperature of 10 °C, as a better tool for forecasting their harvest periods than relying on an fixed number of days between bud break and ripening. Climate change acceleration makes old calculations based on counting the calendar days required from bud break to harvest no longer useful in grapevine [10]. On the contrary, global warming projections indicate the anticipation of the phenological events and that plants will fulfill their heat requirements in a shorter period [11]. Climate changes also makes more difficult forecasting harvest dates [12] and planning the cultivation techniques [13]. An accurate estimation of heat requirements based on cardinal temperatures regardless the time required for its fulfillment can be helpful in this context [14]. Verdugo-Vásquez et al. [15] found strong nonlinear correlations between heat unit accumulation and phenology in four reputed table grape varieties (“Crimson Seedless”, “Superior Seedless”, “Thompson Seedless” and “Red Globe”) and defend that phenological models based on the satisfaction of heat requirements between phenophases, especially from bud break to bloom, can be an useful tool for table grape cultivation.

The precise knowledge of the heat requirements of a given cultivar is even more necessary for table grape cultivation under plastic, since the strong modification of plant phenology imposed by the greenhouse makes it largely inappropriate to estimate the cycle length by counting calendar days; that is, the time measured in days between two distinct phenophases. On the contrary, for a plant genotype, heat requirements measured as growing degree days (GDD) are assumed to be fairly constant in different seasons and sites as long as its cultivation is not negatively affected by any kind of stress [16,17] that could limit its growth and development. Even so, the precise estimation of GDD required for the transition between phenophases of a given genotype depends on the accurate determination of its base temperature (T_b_) and of its upper threshold temperature (T_u_). The lower developmental threshold for a species or base temperature is the temperature at and below which development stops. Upper threshold temperature, also known as upper development threshold, is less well defined, but is often taken as the temperature at and above which the rate of growth or development begins to decrease [18]. Although less well defined, for some cereal and horticultural crops, temperature upper threshold temperatures have been determined and found useful in hot environments [19].

This work aims to determine the lower (T_b)_ and upper (T_u_) threshold temperatures for “Flame Seedless” table grape and to establish the heat unit requirements of this cultivar from bud break to ripening in order to improve crop management under a greenhouse, making it possible to suggest the appropriate timetable for cultural practices such rest breaking agents application, berry and cluster thinning, and harvest.

## 2. Results

### 2.1. Cardinal Temperatures for “Flame Seedless”

All assessed methods (SD in GDD and days, CV in GDD, RE and x-intercept) provided a first estimation of T_b_ values that were plausible and compatible with “Flame Seedless” table grape development (Table 1 first line). Nonetheless, the proposed values of the different methods did not coincide at all. The least SD method, both in GDD and in days, gave T_b_ values close to 16 °C, while CV in GDD, RE and x-intercept methods coincided, showing T_b_ values between 6 and 7 °C. When we checked the robustness of the methods by removing the data of the coolest and of the warmest seasons (2005 and 2006, respectively), the values for T_b_ became extremely variable, assigning in some occasions unrealistic results for T_b_ (Table 1 second line).

**Figure 1:**
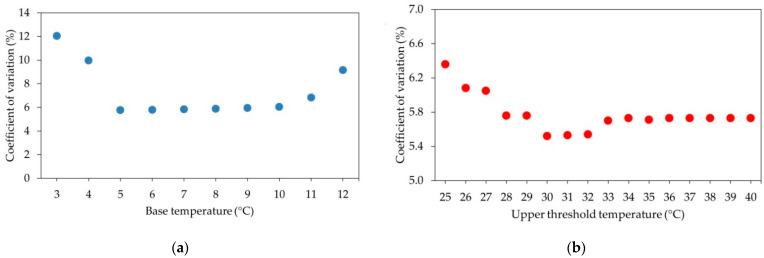
Coefficient of variation (CV) in growing degree days (GDD) for the period between bud break and harvest at various base temperatures (**a**) and upper threshold temperatures (**b**). Data set from five seasons.

Given this inconsistency, and according to the strong preference shown by Arnold [20] and Perry et al. [21] for the method based on the least CV in GDD, we checked the T_b_ value that presented the least CV in the estimations of GDD between bud break and ripening in the range of temperature of 3–12 °C, proposed as likely T_b_ by different authors [22,23,24,25]. Figure 1a shows how the CV of GDD estimations diminishes very slightly as the temperature falls between 10 and 5 °C. On the contrary, the CV of GDD rose considerably beyond these limits. The least CV (5.77%) was found at 5 °C and, therefore, this value is proposed as T_b_ for “Flame Seedless”.

We used the same procedure for calculating T_u_, this time in the range of 25–45 °C. In this case, the least CV in GDD was obtained for T_u_ = 30 °C (5.52%) (Figure 1b). Temperatures above 33 °C all coincided in producing a slightly higher CV value (5.70%).

### 2.2. Determination of the Heat Requirements of “Flame Seedless” from Bud Break to Ripening—Accuracy and Robustness

**Figure 2:**
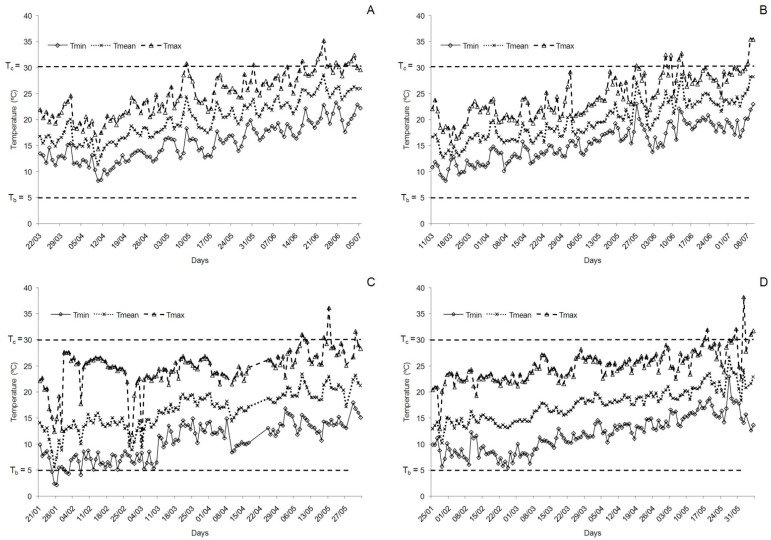
Minimum, mean and maximum daily temperatures during the coolest season (2005) and the warmest season (2006) in the open field (**A** and **B**, respectively) and inside the plastic greenhouse (**C** and **D**, respectively).

“Flame Seedless” table grape required an average of 1633 GDD from bud break to ripening with T_b_ = 5 °C and T_u_ = 30 °C. In our latitude, these requirements were completed from March to July in an average of 117 days in the open field (Table 2). This calculation allows forecasting “Flame Seedless” harvest date in open-field-grown vines with a mean accuracy of three days. The greatest error was obtained in the coolest season of 2005, when the prediction deviated eight days from the actual date of harvesting. A difference of six days was also noted between the predicted and the observed harvest dates in 2006 (the warmest season) (Table 3). The time course of minimum, mean and maximum daily temperatures during these two seasons is depicted in Figure 2. Remarkable accuracy was obtained in the remaining three seasons (Table 3). Despite the cited deviations, GDD summation provided in all seasons a more accurate forecast of harvest date than relying on the average number of days between bud break and ripening. The mean error in days when harvest prediction was based on the average number of days was also higher than the error when the prediction relied on GDD summation (five days for calendar days versus three days for GDD summation) (Table 3). The greatest error committed by the method based on the average number of days between bud break and ripening occurred again in 2005, when a difference of 13 days was observed between the predicted and the observed harvest dates (Table 3).

After comparing the different methods for establishing T_b_ and T_u_, making the calculations and checking the deviation between the predicted and the observed harvest dates, we used season 2009 data to check the robustness of the calculations and its accuracy to predict harvest date with a reasonable exactitude in both environments. The ability of the procedure based on heat unit requirements using the average year was found quite satisfactory. In the season 2009, the procedure predicted the beginning of harvest on 12 July 2009, while the berries reached the maturation index established for harvesting at 16 July 2009; that is, four days later than predicted. If we used the typical meteorological year instead of the average year, then the prediction ability was greatly improved since harvest was forecast at 15 July 2009; that is only one day before harvesting. The method of adding calendar days from 2009 bud break date to ripening was again less reliable, miscalculating harvest commencement by six days.

### 2.3. Harvest Prediction in “Flame Seedless” Table Grape Cultivated under Greenhouse

The ability of the previous GDD value to predict harvest date accurately in protected cultivation was, on the contrary, less satisfactory. The mean accuracy in predicting harvest date in “Flame Seedless” cultivated under greenhouse was six days (Table 3). It is noteworthy that in all cases the procedure predicted a harvest date some days later than the actual one. In other words, the actual beginning of harvesting always came earlier than expected. This was the result of a lower accumulation of heat units required between bud break and ripening in the greenhouse (1542 GDD) than that observed for the same transit between these phenophases in the open-field-grown vines (1633 GDD).

As for open field vines, the greatest error occurred in 2005, when the prediction miscalculated the actual harvest date in the greenhouse by 10 days. The error was of six and eight days for 2007 and 2008, respectively. In 2006, the error was of just one day (Table 3). Under all circumstances, harvest prediction was more accurate using GDD estimations than estimations based on the average number of days (Table 3). In fact, the mean error when the prediction was based on calendar days was much higher than when the prediction relied on GDD (14 days versus 6 days) (Table 3). In contrast with the estimation based on GDD, the method based on the average number of days from bud break to ripening always predicted the beginning of harvesting well before the observed date. In this regard, a risk for premature harvesting exists when relying on the estimation based on calendar days; for grapes, the consequences are very negative and relevant in the quality of this nonclimacteric fruit. The prediction ability of the procedure for the following season (2009) was more satisfactory, predicting the harvest on 6 June, four days earlier than the date when maturation index reached the threshold value of 18 (10 June 2009).

## 3. Discussion

The heat requirements of “Flame Seedless” table grape between bud break and ripening have been estimated as an average of 1633 GDD for T_b_ = 5 °C and T_u_ = 30 °C. A much higher heat requirement for “Flame Seedless” is proposed by Menora et al. [26] in semiarid tropical conditions of Southern India. The main source of the error is that these authors calculated the required GDD (and days) after winter pruning date as starting point (that can be widely variable) and not from bud break. These authors chose 10 °C as base temperature but did not use an upper threshold temperature for their calculations.

Our estimation of heat requirements allowed forecasting “Flame Seedless” harvest date accurately with an error of just three days. The procedure based on GDD estimations clearly improved the prediction of harvest date based on calendar days (Table 3), as Van Den Brink [27] observed for “Concord” (*V. labrusca*) grape and Williams et al. [28] for “Thompson Seedless” (*V. vinifera*). This improvement in forecasting harvest date using GDD is of great utility for programming the marketing and commercialization of the oncoming yield, and also for minimizing consumers’ risks relative to mistimed phytosanitary applications when harvest date is projected with such precision. Our preference for GDD is in agreement with Zhou and Wang [29], who used a nonlinear method for the calculation of GDD in two cereals (wheat and corn). These authors emphasize that the use of GDD has improved the prediction of phenological events (here, berry ripening) compared with other approaches, such as the time of year or the number of days elapsed from a starting point or phenological event. Other authors observed, however, better performance counting the number of days between phenophases in more predictable climates (Minas Gerais and Sao Paulo, Brazil) [30,31,32]. This system is simpler but presumably less accurate and of less utility for other sites and climates.

The greater accuracy of the GDD method for estimating harvest date of “Flame Seedless” was more obvious in protected cultivation, where the length of the cycle of “Flame Seedless” was 13 days longer on average than in the open field. This longer duration of the cycle is mainly due to the long time taken from bloom to harvest inside the greenhouse (Table 2), and more specifically from veraison to harvest. As Jones and Davis [33] observed in wine grape cultivars, longer intervals between events indicate less than ideal climate conditions and a delay in growth and maturation, as we have noticed in the greenhouse. This adversity does not completely cancel the improvement in fruit earliness carried out in the greenhouse, since the beginning of the cycle was advanced by protected cultivation into a greater extent, bringing bud break to the month of January. Indeed, the success of protected cultivation of early maturing table grapes relies on the advancement of bud break dates under greenhouse [2,5], although part of the earliness was lost in phenophases between bloom and berry ripening [2,3], as occurred here.

The ability of the procedure developed here to predict accurately harvesting relies on a precise determination of T_b_. For this determination, we evaluated five mathematical methods proposed by Arnold [20], finding major inconsistencies in all of them when we modified the number of experimental seasons (Table 1). A similar inconsistency in determining T_b_ for the wine cultivar “Touriga Francesa” was reported by Oliveira [24]. Given this situation, we chose, according to Arnold’s preference [20], the T_b_ value that presented the least CV in the estimations of GDD, namely T_b_ = 5 °C. This temperature (5 °C) is lower than the one usually reported in the literature (10 °C) [8,9,27,33,34,35]. Under different climatic conditions, however, the base temperature seems to differ from 10 °C [22,36] and we found that the use of T_b_ = 5 °C versus 10 °C improved harvest date prediction under protected cultivation (a mean error of six days for T_b_ = 5 °C versus 14 days when using T_b_ = 10 °C). Our proposal coincides with Molitor et al. [37], who established a cumulative degree day model to simulate phenological development of grapevine based on long-term data sets from six locations in four European countries and obtained the best results using also a base temperature of 5 °C. García de Cortázar-Atauri et al. [38], working with ten cultivars of grapevine in five different locations, did also show a preference for a base temperature of 5 °C in comparison to T_b_ = 10 °C. Moncur et al. [22] suggest 4 °C or less as the base temperature and indicate, as Oliveira [24] did, that there are no experimental reasons behind the common proposal of T_b_ = 10 °C in *V. vinifera*. Schrader et al. [39] indicate T_b_ = 10 °C may be a poor predictor of bloom, and bud break phenology, during years with uncommon conditions such as an early spring, as we found while cultivating “Flame Seedless” in a greenhouse. Several reports have documented, however, that photorespiration in grape is almost nil below 10 °C [40,41]. Bonhomme [16] underlines that the base temperature has often only statistical value, and usually is quite distant from the physiological temperature for which plant development is zero.

The establishment of an upper threshold temperature is not common in viticulture. However, we found an improvement of two–three days in our predictions in some seasons when we included 30 °C as T_u_. This does not imply that plant development is totally arrested at this point. Indeed, we are aware that gas exchange in “Flame Seedless” is not completely inhibited at this temperature [42]. Nonetheless, limiting plant heat accumulation above certain limits (in our case 30 °C) improved our predictions, as McIntyre et al. [35] indicate. Greer [43] analyzed net assimilation response of variety “Semillon” and found maximum levels of photosynthesis between 25 and 30 °C. This latter value (30 °C) coincides with the experimentation carried out by Kriedemann and Smart [44], who observed that photosynthesis showed an optimum near 30 °C, and a sharp decline at higher temperatures, due to stomatal closure, when leaf moisture tension approached 15 bar. Kriedemann [45] found that optimum temperature for net photosynthesis was 25 °C for greenhouses vines and 30 °C for fully sun-exposed vines grown in the open field, with a sharp decline of net assimilation above 35 °C. Ferrini et al. [46] also measured in greenhouses experiments a substantial diminution of the photosynthetic activity in “Trebbiano Toscano” vines grown at 35 °C in comparison to plants grown at 27.5 °C, suggesting T_u_ is established between these two values. Finally, Molitor et al. [37] followed the same reasoning but proposed 22 °C as the temperature above which net assimilation is negative in grape.

During our experimental seasons, temperature rarely dropped 5 °C or exceeded 30 °C. When they did, it was mostly at the beginning of the year (for T_b_) and at end of the season for T_u_ (Figure 2). In any case, we have to indicate that the changes of the CV (%) in GDD in the range of 30–40 °C proved minimal (Figure 1b); hence T_u_ = 35 °C proposed by Buttrose and Hale [47] and Kadir [48] as the limiting temperature for bloom and subsequent fruit set and development would have provided similar accuracy. In fact, the values of T_b_ and T_u_ changed slightly when we simultaneously determined them by pairing values. Using this approach, the CV was reduced from 5.52% to 5.30% for values of T_b_ = 7 °C and T_u_ = 31 °C. This small reduction of CV did not improve the capacity of harvest prediction that actually worsens in both growing conditions (open field and protected cultivation), and also when we forecast harvest date under the greenhouse in season 2009 (data not shown).

The usefulness of the method was proved acceptable and its prediction capacity satisfactory. However, the poor prediction found in the coolest and warmest seasons of 2005 and 2006, respectively, suggests a limited usefulness of the method (though it is always preferable to estimations based on the number of days between bud break and ripening) for seasons in which the weather substantially differs from the average year. Mauromicale et al. [49] and Perry and Wehner [50] coincide in the limited utility of the GDD procedure to predict accurately phenological events in contrasting environments. On the contrary, the estimation of season length based on calendar days has similar or even greater utility than the GDD method in places enjoying predictable weather [50,51,52,53].

The greater accuracy for the vines grown in the open field than for those grown in the greenhouse (a mean error of three days versus six days) appears to be due to the seemingly lower heat units that “Flame Seedless” vines required to reach maturation under the greenhouse. These differences in GDD needs were more apparent between bud break and bloom (Table 2), a period when vines are more affected by ambient temperature than the period from bloom to ripening, considered more genotype-dependent [54,55]. The easiest explanation of this fact is that vines in the greenhouse developed more efficiently in thermal terms, and this is possible because the setting of temperatures within the greenhouse provided optimum temperatures for longer periods, so plants development might occur at higher rates during more time, confirming once again that plant response deviates from linearity [20,56]. This is especially true at the extremes of the temperature range and especially near harvest when farm management has a great impact on vine phenology [57]. In spite of the error performed by using linear equations, more complicated calculations brought by the use of complex nonlinear equations often results in only minor improvements in the predictions [16,37]. Further improvement in our prediction was achieved when we recalculated the heat unit needs of “Flame Seedless”, taking into consideration the average GDD observed in the greenhouse rather than that computed in the open field (Table 4).

Finally, we are aware that, by theory, the same heat requirement amount is expected for a given genotype regardless of the cultivation site. However, for this to be true, plants have to be under good management and free of resource limitations and of any kind of stress that might delay berry growth and ripening. On the other hand, most plant processes (photosynthesis, respiration, water uptake, growth) follow sigmoid curves, with a linear approach an approximation to the central part of that sigmoid curve. Thus, the occurrence of extreme values far from the optimum might represent a source of error in the correct estimation of heat requirements. It is also important to explain that despite the lower heat unit needs of “Flame Seedless” in the greenhouse than in the open field, the cycle of “Flame Seedless” was longer under protected cultivation. Simply put, the vines need more days to satisfy lower heat requirements. This apparent paradox is linked to the phenological displacement to earlier dates brought by protected cultivation. In other words, forcing a table grape crop to initiate development in January has a cost to pay, although the price is worth it when the earliness achieved allows “Flame Seedless” table grape commercialization in June.

## 4. Materials and Methods

### 4.1. Site and Plant Management

This study was performed in a “Flame Seedless” vineyard located at the Cajamar Foundation Research Center, in El Ejido (Almería, SE Spain) (longitude 2°43′10″ W, latitude 36°47′40″ N). The altitude is 151 m above sea level and the vineyard is 11 km away from the Mediterranean Sea. The experimental area presents a semiarid subtropical Mediterranean climate according to the agroclimatic classification of Papadakis [58], with an average annual temperature around 18.5 °C. December and January are the coolest months and August the warmest. Rain averages 250 mm per year (January to December), while mean annual relative humidity oscillates between 67% and 73% depending on the year. Bright sunny days are common at the experimental site. Sunlight hours reach a mean value of 3273 h per year. The soil is a sandy clay loam with 49.6% sand, 26.4% silt and 24.0% clay, measured at 10–70 cm depth, where most roots of the vines grow.

The “Flame Seedless” vines used for this study were planted in 1999, grafted on 161-49 C rootstock. The vines were arranged on a 3.5 × 3.5 m spacing and trained according to local practices in a 2.1 m high Spanish trellis system (“parral”). The vineyard was divided into two plots (800 m^2^ per each), one located under a greenhouse structure and the other in the open. The greenhouse was a flat roof structure covered with a three-layer polyethylene plastic film of 0.2 mm thickness, situated 1.40 m above the vine canopy. This greenhouse had two side vents (in the north and south walls, respectively) and four alternately oriented roof flaps vents (west–east) that remained completely open until December of each experimental year to allow vines to fulfill their chilling requirements. Chilling requirements of “Flame Seedless” are estimated in around 150 h below 10 °C by the Utah model of chill units [59]. The opening and closing of the vents was afterward automated and controlled by a climate control system (Mithra Clima, Priva Nutricontrol Ibérica S.L, Cartagena, Spain). The temperature for activating the opening of the windows of the greenhouse was established at 16 °C from bud swelling to bloom, and increased up to 20 °C for the rest of the cycle according to the suggestions made by Colapietra [60]. Daily thermal amplitude was, in spite of the automated opening of the windows, commonly wider within the greenhouse than in the open field (around 15 °C in the greenhouse versus 10 °C in the open field).

Crop load was mainly regulated by pruning, leaving 9–10 canes per vine each one bearing 10 buds, and 4 spurs to form new canes as the replacement for the production of the next year yield. Pruning was performed at the beginning of December in the vines inside the greenhouse, and one month and a half later in the open field. Crop load was later more precisely adjusted by cluster thinning at prebloom, leaving between 50 and 80 clusters per vine depending on the year as the vine aged. Canes were bent to enhance the percentage of buds sprouting. Hydrogen cyanamide was also applied on greenhouse vines at a dose of 5% to increase and advance bud break. This application was performed in mid-December, according to programs derived from previous experiences. Hydrogen cyanamide was not needed in the open field where bud break takes place normally in the experimental site.

The phenology of the experimental vines was monitored every week starting at bud swelling. The examination was carried out by trained technicians on all buds of 3 canes per vine and 9 vines per plot (open field and greenhouse) according to the BBCH scale [61]. Special attention was paid to the determination of bud break (BBCH stage 09; green tissue seen between bud scales), full bloom (BBCH stage 65; 50% of open flowers) and ripening (BBCH stage 89; beginning of harvest). Bud break and full bloom dates were established when 50% of the total buds of the sampled canes in each vine reached these phenological stages [62]. Harvest date was established as the first day we collected clusters where berries had reached a maturity index above 18 [63]. Berry sampling was performed weekly from veraison to ripening to establish this moment. This monitoring was done on a sample of 60 berries per replicate (20 per vine), berries collected from as many colored bunches as possible from each vine from all parts of the bunch (tail, center and shoulders). In the laboratory, the must obtained from each sample, was centrifuged and total soluble solids measured (and expressed as °Brix) with a Shibuya refractometer (Shibuya Optical Co., Ltd., Wako-shi, Japan). Acidity was measured by titration with 0.1 N NaOH to pH 8.2. The maturation index was calculated as the ratio between total soluble solids (measured as °Brix) and titratable acidity (expressed as g/L of tartaric acid) [63].

All vines were grown under nonlimiting conditions of watering and fertilizers and maintained pest-free according to the practices in the area. The crop load was also adequate with yields close to 23 t ha^−1^, without significant differences between growing conditions and among seasons, with the exceptions of season 2009, when vines in the open field produced significantly more yield than plants grown in the greenhouse due to much heavier clusters (741 versus 343 g, in the open versus greenhouse, respectively).

### 4.2. Threshold Temperatures for “Flame Seedless”

The first step of this study was to determine “Flame Seedless” base temperature (T_b_). To do so, we compared five mathematical methods proposed by Arnold [20]. These five methods are the least standard deviation (SD) in GDD, the least SD in days, the least coefficient of variation (CV) in GDD, the regression coefficient (RE) and the x-intercept. The reliability of different T_b_ values projected by the methods cited above was assessed by their consistency in different years and by their compatibility with *V. vinifera* growth. The mathematical formulae of four of the methods (x-intercept not included) are described by Yang et al. [64]. The upper threshold temperature (T_u_) value was then chosen using the previously selected method. This calculation was performed over a temperature range of 25–45 °C given that different authors have proposed that T_u_ ranges from 30 to 40 °C [33,35,47,54,55,65]. Hourly temperatures at the open air were retrieved from a weather station located at the Research Center, 200 m from the experimental plot, for the determination of the threshold temperatures. 

### 4.3. Growing Degree Days Determination—Accuracy and Prediction Ability of the Procedure

Next, the heat requirements of “Flame Seedless” from bud break to ripening were calculated as the average of the GDD computed over five seasons (2003 and from 2005 to 2008) between these two phenological stages, in the vines cultivated in the open field. The calculation of GDD was carried out each year taking into consideration the T_b_ and T_u_ values previously established, and the hourly temperature records during the bud break–ripening period recorded at the same weather station following the next Equation (1):

(1) $$\mathrm{GDD}=\overset{n}{\underset{i=1}{\sum }}\left(\frac{\mathrm{GDH}i}{24}\right)$$

where GDD is the growing degree days accumulated; GDH*i* is the growing degree days accumulated each hour and *i* is the day between bud break and harvest. GDH*i* was computed according to the following conditional Equation (2):

(2) $$\mathrm{GDH}i=\overset{24}{\underset{h=1}{\sum }}\left(T_{h}\;-\;T_{b}\right)$$

where T_h_ is hourly temperature. If T_h_ ≤ T_b_, then GDH*i* = 0; if T_b_ < T_h_ ≤ T_u_, then we use Equation (2); if T_h_ > T_u_, then GDH*i* = T_u_ − T_b_.

The accuracy of the GDD determination and the prediction ability of the procedure were then checked. The accuracy of the average GDD value was assessed by comparing the predicted and the observed harvest dates for the five seasons for which these phenological data were available, considering the real temperatures measured each year in the plot. The prediction ability of the procedure was, on the other hand, assessed by projecting the harvest date for the coming season. In this analysis, we forecast the harvest date for the 2009 season by projecting previous GDD calculations on an average year and on a typical meteorological year (TMY). Bud break date of season 2009 was considered as the starting point [37]. The average year was built by Fernández et al. [66] based on the hourly course of temperatures in the open air at the experimental site from 1991 to 2010, an optimum lapse period for our 2009 harvest prediction. TMY was built selecting the most representative months of the year by analyzing the time series of the same lapse period (1999–2010) at the experimental site. To generate this TMY, Fernández et al. [66] checked three different methodologies (Sandia National Lab, Pissimanis and Argiriou) and concluded that the method proposed by Argiriou et al. [67] gives the best results for our experimental site.

### 4.4. Harvest Prediction in “Flame Seedless” Table Grape Cultivated under Greenhouse

The robustness of the procedure to forecast accurately the harvest date in protected cultivation was also checked by comparing the predicted and the observed harvest dates for four seasons (from 2005 to 2008) taking into consideration the temperatures measured in the greenhouse each year. These temperature data were retrieved from measurements taken with Pt-100 probes incorporated in an aspirated psychrometer located within the greenhouse. Finally, we forecast the 2009 harvest date for “Flame Seedless” vines cultivated under greenhouse by applying the previous GDD calculation on an average year, considering bud break in the 2009 season as the starting point. In this case, the average year was based on the records of the daily evolution of temperatures within the greenhouse (five years of data available).

## 5. Conclusions

Determining the cardinal temperatures (T_b_ = 5 °C and T_u_ = 30 °C) for “Flame Seedless” table grape allowed us to predict its ripening with an error of just three days, thus improving our capacity to forecast harvest date in comparison with the usual method based on counting the number of days between bud break and ripening. The improvement in harvest date prediction was better for “Flame Seedless” cultivated in a greenhouse, where the annual cycle was lengthened. The ability of the procedure based on GDD to forecast oncoming harvest date was found quite satisfactory. If we used the typical meteorological year instead of the average year, then the prediction was greatly improved since harvest was forecast just one day before its occurrence. We are aware, however, of the need to update results in future works since global warming can make past estimations based on counting the calendar days between bud break and ripening mostly useless, but less, actually, when we rely on GDD.

## Figures and Tables

**Table 1:** Base temperature (°C) estimation for “Flame Seedless” table grape between bud break and harvest using five different methods and based on different sets of seasons.

Data Sets	SD ^1^_GDD_^2^	SD_DAYS_	CV ^3^_GDD_	RE ^4^	X-Intercept
Five seasons	15.82	15.53	5.94	7.10	6.86
Three seasons (excluding 2005 and 2006)	28.54	357.50	338.73	347.32	105.00
Four seasons (excluding 2005)	35.82	64.22	107.94	108.80	58.50
Four seasons (excluding 2006)	14.71	14.30	7.96	8.66	8.37

^1^ SD: standard deviation; ^2^ GDD: growing degree days; ^3^ CV: coefficient of variation; ^4^ RE: regression coefficient.

**Table 2:** Estimated heat requirements measured in growing degree days (GDD) with T_b_ = 5 °C and T_u_ = 30 °C, and season length duration in days of “Flame Seedless” grapevines cultivated in open field and in a plastic greenhouse.

Open Field						
Season	Bud Break–Full Bloom		Full Bloom–Harvest		Total	
	GDD	Days	GDD	Days	GDD	Days
2003	679	62	974	59	1652	121
2005	628	52	862	52	1490	104
2006	620	54	1120	68	1740	122
2007	703	65	932	55	1634	120
2008	841	71	808	48	1650	119
Mean	694	61	939	56	1633	117
**Greenhouse**						
**Season**	**Bud Break** **–Full Bloom**		**Full Bloom** **–Harvest**		**Total**	
	**GDD**	**Days**	**GDD**	**Days**	**GDD**	**Days**
2005	574	64	924	69	1498	133
2006	641	64	984	68	1625	132
2007	613	58	929	70	1542	128
2008	590	58	913	70	1503	128
Mean	605	61	937	69	1542	130

**Table 3:** Observed and predicted harvest dates for “Flame Seedless” in open field and greenhouse based on GDD and calendar day methods.

Open Field					
Season		GDD		Calendar Days	
	OHD ^1^	PHD ^2^	PHD − OHD	PHD	PHD − OHD
2003	11 July	10 July	−1	7 July	−4
2005	3 July	11 July	+8	16 July	+13
2006	10 July	4 July	−6	5 July	−5
2007	9 July	9 July	0	6 July	−3
2008	14 July	14 July	0	12 July	−2
Accuracy			3		5
**Greenhouse**					
**Season**		**GDD**		**Calendar Days**	
	**OHD** **^1^**	**PHD** **^2^**	**PHD − OHD**	**PHD**	**PHD − OHD**
2005	2 June	12 June	+10	17 May	−16
2006	6 June	7 June	+1	21 May	−16
2007	5 June	11 June	+6	25 May	−11
2008	29 May	6 June	+8	18 May	−11
Accuracy			6		14

^1^ OHD: observed harvest date; ^2^ PHD: predicted harvest date.

**Table 4:** Accuracy in harvest date forecasting for “Flame Seedless” table grape cultivated under greenhouse, based on GDD vs. the calendar day methods.

Season	OHD ^1^	GDD T_b_ 5 °C and T_u_ 30 °C	PHD ^2^	PHD − OHD	Calendar Days	PHD	PHD − OHD
2005	2 June	1498	5 June	+3	133	30 May	−3
2006	6 June	1625	30 May	−7	132	4 June	−2
2007	5 June	1542	5 June	0	128	7 June	+2
2008	29 May	1503	1 June	+3	128	31 May	+2
Mean		1542			130		
Accuracy				3			2

^1^ OHD: observed harvest date; ^2^ PHD: predicted harvest date.

## Data Availability

All tables and figures are original.
